# Discovery of APL-1030, a Novel, High-Affinity Nanofitin Inhibitor of C3-Mediated Complement Activation

**DOI:** 10.3390/biom12030432

**Published:** 2022-03-11

**Authors:** Joshua Garlich, Mathieu Cinier, Anne Chevrel, Anaëlle Perrocheau, David J. Eyerman, Mark Orme, Olivier Kitten, Lukas Scheibler

**Affiliations:** 1 Apellis Pharmaceuticals, Waltham, MA 02451, USA; josh.garlich@apellis.com (J.G.); david.eyerman@apellis.com (D.J.E.); mark.orme@apellis.com (M.O.); 2 Affilogic SAS, 44200 Nantes, France; mathieu@affilogic.com (M.C.); anne@affilogic.com (A.C.); anaelle@affilogic.com (A.P.); olivier@affilogic.com (O.K.)

**Keywords:** alternative scaffold protein, affitin, complement, molecular biology

## Abstract

Uncontrolled complement activation contributes to multiple immune pathologies. Although synthetic compstatin derivatives targeting C3 and C3b are robust inhibitors of complement activation, their physicochemical and molecular properties may limit access to specific organs, development of bifunctional moieties, and therapeutic applications requiring transgenic expression. Complement-targeting therapeutics containing only natural amino acids could enable multifunctional pharmacology, gene therapies, and targeted delivery for underserved diseases. A Nanofitin library of hyperthermophilic protein scaffolds was screened using ribosome display for C3/C3b-targeting clones mimicking compstatin pharmacology. APL-1030, a recombinant 64-residue Nanofitin, emerged as the lead candidate. APL-1030 is thermostable, binds C3 (K_D_, 1.59 nM) and C3b (K_D_, 1.11 nM), and inhibits complement activation via classical (IC_50_ = 110.8 nM) and alternative (IC_50_ = 291.3 nM) pathways in Wieslab assays. Pharmacologic activity (determined by alternative pathway inhibition) was limited to primate species of tested sera. C3b-binding sites of APL-1030 and compstatin were shown to overlap by X-ray crystallography of C3b-bound APL-1030. APL-1030 is a novel, high-affinity inhibitor of primate C3-mediated complement activation developed from natural amino acids on the hyperthermophilic Nanofitin platform. Its properties may support novel drug candidates, enabling bifunctional moieties, gene therapy, and tissue-targeted C3 pharmacologics for diseases with high unmet need.

## 1. Introduction

The complement system is a central component of innate immunity that provides integrated host defense to eliminate foreign or damaged cells [1,2,3]. The complement network comprises over 40 proteins that can be activated via multiple pathways [1,2,4]. Each of the three major complement activation pathways (classical, alternative, and lectin) culminates in the cleavage of the central component, C3, into functional fragments C3a (an inflammation mediator) and C3b (an opsonin) [1,2,4]. Downstream of C3 cleavage, C3b can combine with other complement fragments to generate C5 convertases, which cleave C5 and initiate the formation of the membrane attack complex [1,4,5,6]. 

**Figure 1:**
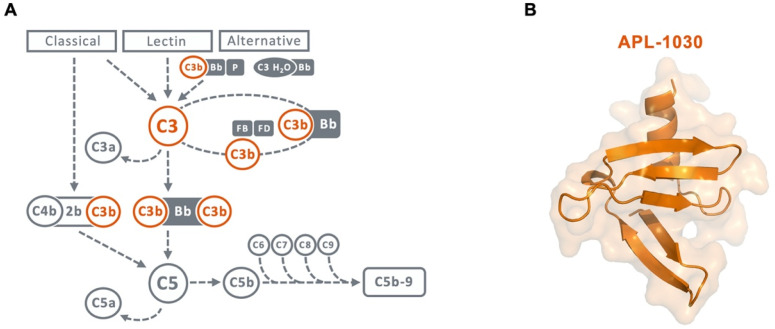
(**A**) Scheme of the complement cascade highlighting the central role of C3 and C3b in the amplification loop. Therapeutic targets for inhibition by APL-1030 are marked in orange. (**B**) Ribbon and surface representation of the 3D structure of APL-1030, with C-terminal alpha helix (**top**) and N-terminal end (**bottom**).

Tight regulation of complement activation is essential for immune surveillance and homeostasis [2,7]. Undesired or unregulated complement activation contributes to the pathophysiology of numerous complex immune diseases, such as C3 glomerulopathies, age-related macular degeneration, and paroxysmal nocturnal hemoglobinuria (PNH), as well as various neurodegenerative diseases of the central nervous system (CNS) and peripheral nervous system (PNS), including Alzheimer’s disease, amyotrophic lateral sclerosis (ALS), and myasthenia gravis [7,8,9,10,11,12,13,14]. From a therapeutic perspective, inhibition of C3-mediated activation is of particular interest in complement-mediated diseases, as all main complement activation pathways converge on C3 (Figure 1A) [15,16,17,18]. Anti-C3b molecules have also demonstrated in vitro disruption of C3b fragment deposition, C3 and C5 convertase formation, and alternative pathway activation [19,20,21]. Taken together, these results suggest a therapeutic benefit of a dual inhibitor targeting both C3 and C3b. Drug candidates that act directly on C3 to inhibit its activation are currently being evaluated in preclinical and clinical settings for complement-related diseases [18,22,23].

Compstatin, a 13-residue synthetic peptide, demonstrates strong affinity for native C3, as well as for fragments C3b and C3c, and inhibits both the classical and alternative pathways of complement activation [15,24]. Compstatin binds C3 at the macroglobulin (MG) domains 4 and 5 within the MG ring, a component of the structurally stable β-chain of C3, as well as its fragments C3b and C3c [25]. The compstatin-binding site on C3 is far from other known binding sites, and its binding does not result in large structural changes to C3, suggesting that compstatin binding sterically hinders C3 binding to convertases [25]. Synthetic analogs of compstatin have demonstrated similar pharmacology in preclinical models and are being evaluated in clinical trials for complement-mediated diseases, including PNH, age-related macular degeneration, hematopoietic stem cell transplantation-associated thrombotic microangiopathy, amyotrophic lateral sclerosis (ALS), and C3 glomerulopathies [18,26,27,28]. In a milestone for complement-mediated diseases, the first C3-targeting modality, pegcetacoplan, a derivative of compstatin, was approved in 2021 for the treatment of PNH, establishing C3 as a generally safe and viable therapeutic target for this condition [29]. Promising results from these trials support the continued investigation of C3/C3b-targeting therapies for other complement-mediated diseases.

The objective of the current study was to identify novel, small protein, biotherapeutic drug candidates that demonstrate high affinity for the compstatin C3-binding site with characteristics that enable recombinant production, improved thermostability, and use in multifunctional constructs to enable poly-pharmacology, half-life extension, and tissue-targeted therapy.

Nanofitins are small alternative protein scaffolds derived from Sac7d, a 66-residue protein isolated from the hyperthermophilic archaeon *Sulfolobus acidocaldarius* [30,31]. The randomization of the natural DNA-binding site of Sac7d generates libraries of Nanofitins, from which high-specificity binders to different molecules can be selected while maintaining their chemical and thermal stability [30,32,33]. Additionally, Nanofitins show promising bifunctional therapeutic potential because their N- and C-termini ends reside on opposite faces of their variable domain, allowing multispecific assemblies using straightforward molecular approaches, while preserving the original pharmacologic and stability properties of the parent protein [30,31,32]. In addition, the small size of Nanofitins (7 kDa) [31], their thermostability [34], and their potential for transgenic expression to enable gene therapy-based delivery [35] may allow for targeted complement modulation in the brain, eye, muscle, and other tissues of interest, offering novel therapeutic opportunities. Here, we describe the discovery and in vitro characterization of APL-1030 (Figure 1B), a novel, high-affinity Nanofitin C3/C3b binder and complement system inhibitor.

## 2. Materials and Methods

### 2.1. Biotinylation of Antigens

C3 and C3b (Complement Technology, Inc., Tyler, TX, USA) were biotinylated and used for the selection and identification of clones. The target protein (50 µM) was incubated with a 5-fold molar excess of sulfosuccinimidyl-6-(biotinamido) hexanoate (Sulfo-NHS-LC-LC-Biotin) (Pierce Biotechnology, Rockford, IL, USA) in phosphate-buffered saline (PBS) (Sigma-Aldrich, St. Louis, MO, USA) on ice for 1 h. Then, protein-desalting spin columns equilibrated in Tris-buffered saline (TBS) (20 mM Tris–HCl, 150 mM NaCl, pH 7.4; MilliporeSigma, Burlington, MA, USA) were used to buffer-exchange biotinylated protein. The 4′-hydroxyazobenzene-2-carboxylic acid assay (Sigma-Aldrich) was used to determine the degree of biotinylation. Each protein molecule contained ~2 molecules of biotin.

### 2.2. Ribosome Display Selection Rounds and Isolation of Clones

The combinatorial-naïve Nanofitin library was prepared with minimal modification to a previously described protocol [30,36]. To generate the library, degenerated oligonucleotides using trimer codon mix encoding for all naturally occurring amino acids except cysteine were used in 2 successive overlapping polymerase chain reactions (PCRs). A final PCR step added the 5′- and 3′-flanking regions necessary for ribosome display. The PCR-amplified library was transcribed for selection [36,37]. The focused library designed for affinity maturation was created similarly, using degenerated oligonucleotides encoding NHH triplets (N = A, C, T or G; H = A, C, or T).

Ribosome display selection was performed at 4 °C following a previously described protocol [36,37]. For the initial selection, 4 rounds of selection were performed to isolate high-affinity binders. The pressure of selection was gradually increased in each round by increasing the time in wash steps as follows: round 1, 8 washes of 20 s; round 2, 8 washes of 3 min; round 3, 8 washes of 15 min; and round 4, 4 washes of 15 min followed by 4 washes of 30 min.

Following screening, a full alanine scan of the hit candidate-binding site was performed to identify residues that are critical for C3 specificity. Residues that were determined to not be critical were randomized again to create a focused library. Three rounds of selection were performed for affinity maturation involving a wash-steps scheme, with round 1 consisting of 8 washes of 15 min, and rounds 2 and 3 consisting of 4 washes of 15 min, followed by 4 washes of 30 min. An off-rate pressure of selection was added on rounds 2 and 3. The biotinylated target (10 nM) was co-incubated with the peptides for 1 h, then 1000-fold excess of unbiotinylated target (10 µM) was added and incubated for an additional 1 h (round 2) or 4 h (round 3).

To monitor the enrichment of the diversity toward C3-specific binders, biotinylated target protein (C3 or C3b) immobilized on a streptavidin-functionalized Nunc MaxiSorp (Sigma-Aldrich) plate (Thermo Scientific, Waltham, MA, USA) was incubated with 100 µL of translated pool resulting from each round of selection. For this, an RGS-His antibody horseradish peroxidase conjugate (Qiagen, Hilden, Germany), which detects the RGS-(His)6-tag on the Nanofitins, along with an enzyme-linked immunosorbent assay (ELISA), were used (further details on the ELISA procedure are provided in the ELISA methods section).

Amplified DNA material from the fourth (naïve library) and the third (focused library) round was cloned between BamHI and HindIII restriction sites of a custom derivative of the pQE30 expression vector (pAFG12; Qiagen). This allowed the expression of the Nanofitin with AcGFP fused at its C-terminus using the linker sequence GSAGSAAGSGEF. The ligation mixture was transformed into *Escherichia coli* DH5α LacIq strains (Invitrogen, Waltham, MA, USA). Clones were selected on 2-YT free-medium plates containing 100 µg/mL ampicillin and 25 µg/mL kanamycin (VWR, Radnor, PA, USA). These clones were then inoculated into a deep-well plate holding 0.75 mL of 2-YT medium, which contained 100 µg/mL ampicillin, 25 µg/mL kanamycin, and 1% glucose in each well. Overnight cultures were grown at 37 °C with shaking at 600 rpm. The culture was used to inoculate in mirror a 96-well agar plate supplemented with ampicillin by depositing 10 µL of each culture/well and further processed by Eurofins for plasmid prep and sequencing using the oligo Qe30 for: CTTTCGTCTTCACCTCGA (Plateseq Service, Eurofins genomic, Ebersberg, Germany). A volume of 0.2 mL of each same culture was used to inoculate another deep-well plate containing 1.25 mL of 2X YT medium supplemented with 100 µg/mL ampicillin, 25 µg/mL kanamycin, and 0.1% glucose per well. The plate was incubated with shaking at 600 rpm at 37 °C for 3 h. The addition of 50 µL of isopropyl β-D-1-thiogalactopyranoside (Sigma-Aldrich) at a final concentration of 0.5 mM and incubation with shaking at 600 rpm at 30 °C for 4 h induced the expression of the Nanofitin clones. Cells were pelleted by centrifugation (20 min at 2000× *g*) and retained. Protein extraction was performed with 100 µL/well of BugBuster protein extraction reagent (Novagen, Madison, WI, USA) with shaking for 1 h at room temperature, then 350 µL of TBS was added. Cell debris were pelleted by centrifugation (20 min at 2000× *g*) and screening was performed on supernatants.

### 2.3. ELISA Methods

Streptavidin (66 nM, 100 μL/well; Sigma-Aldrich) was coated on a Nunc MaxiSorp plate by overnight incubation in TBS at 4 °C. Unless otherwise specified, the following steps were performed at room temperature, with shaking at 600 rpm for incubation steps. After 3 washes with 300 µL of TBS, wells were blocked with 300 μL of 0.5% bovine serum albumin (BSA; Sigma-Aldrich) in TBS for 1 h. The blocking solution was removed and biotinylated target proteins (100 μL, 40 nM) in TBS with 0.5% BSA were added and incubated for 1 h. Before each of the following incubation steps, the wells were washed 3 times with TBS with 0.1% Tween 20. Either 100 µL of the crude *E. coli* extracts (ELISA screen) or 100 µL of Nanofitin solutions at increasing concentrations in TBS with 0.1% Tween 20 (half-maximal effective concentration [EC_50_] from 10^−5^ M to 10^−12^ M, with 10-fold dilution) was applied to wells with and without immobilized antigen for 1 h, with shaking. To visualize binding, 100 µL of RGS-His antibody horseradish peroxidase conjugate (Qiagen) diluted 1:4000 in TBS with 0.1% Tween 20 was added and incubated for 1 h with shaking, and then 100 µL of a solution of *o*-phenylenediamine dihydrochloride substrate (Sigma-Aldrich) in revelation buffer (0.05 M citric acid, 0.05% hydrogen peroxide) was added. Absorbance at 450 nm was measured using a Varioskan ELISA plate reader (Thermo Fisher Scientific, Waltham, MA, USA).

### 2.4. Cloning in pQE30 Vector

Subcloning in pQE30 vector was performed by the Gibson assembly method [38]. The pQE30 vector (Qiagen) and the Nanofitin-coding sequence initially embedded in pAFG12 were amplified by PCR using the pairs of oligonucleotides Gpls01-C (TAATGACTGAGCTTGGACTCC) and Gpls01N-Rev (GTGATGCGATCCTCTCATAG), or Gpls01Nsh-Fwd (CTATGAGAGGATCGCATCAC) and Gpls01C-Rev (GGAGTCCAAGCTCAGTCATTAATTAAGCTTTTTCTCGCGTTCCGC), respectively (Eurofins). Linearized vector (100 ng) was mixed with 3 molar equivalents of the gene insert in a final volume of 5 μL. Then, 15 μL of the Gibson assembly mix (25% PEG-8000 [MilliporeSigma], 500 mM Tris-HCl [MilliporeSigma], 50mM MgCl2 [MilliporeSigma], 50 mM dithiothreitol [MilliporeSigma], 1 mM Mix dNTPs [Thermo Fisher Scientific], 5 mM nicotinamide adenine dinucleotide [New England BioLabs, Ipswich, MA, USA], 2 U of T5 exonuclease [New England BioLabs], 12.5 U of Phusion polymerase [New England BioLabs], 2000 U of Taq ligase [New England BioLabs]) were added and the solution was incubated for 1 h at 50 °C. *E. coli* DH5α LacIq strains were transformed with 10 μL of the resulting material. Clones were selected on 2-YT medium plates containing 100 μg/mL ampicillin and 25 μg/mL kanamycin and further validated by Sanger sequencing (GATC Biotech, Constance, Germany).

Constructions of alanine scan variants followed a similar procedure, but the Nanofitin-coding sequence was constructed by 2 successive overlapping PCRs, as reported for the construction of the library. 

### 2.5. Expression and Purification of Nanofitins 

Nanofitins were expressed in *E. coli* DH5α LacIq strains. For this, precultures were grown at 37 °C overnight in 2-YT medium with 1% glucose, 100 μg/mL ampicillin, and 25 μg/mL kanamycin. Dilutions (1:20) of precultures in 2-YT medium with 0.1% glucose, 100 μg/mL ampicillin, and 25 μg/mL kanamycin were grown at 37 °C to mid-log phase (OD 600 = 0.8–1.0). Then, isopropyl β-D-1-thiogalactopyranoside was added to a final concentration of 0.5 mM to induce protein expression and the culture was shaken at 30 °C overnight. Bacteria were pelleted by centrifugation at 3220× *g* for 45 min. A lysis buffer (1X BugBuster Protein Extraction Reagent, 5 μg/mL DNaseI [MilliporeSigma], 20 mM Tris, 500 mM NaCl [MilliporeSigma], and 25 mM imidazole, pH 7.4 [MilliporeSigma]) was used to resuspend cell pellets for cell lysis at room temperature for 1 h. Cell debris was removed by centrifuging the suspension at 3220× *g* for 45 min. Immobilized metal ion affinity chromatography (IMAC) using His60 Nickel Superflow resin (Clontech Laboratories, Mountain View, CA, USA) and a pH 7.4 elution buffer composed of 20 mM Tris, 500 mM NaCl, and 250 mM imidazole was used to purify His-tagged proteins from supernatants. Finally, samples were dialyzed against PBS, filtered on Minisart hydrophilic membranes with 0.2 μm pore size (Sartorius, Bohemia, NY, USA), then stored in sterile conditions at −80 °C. When used in the Wieslab assay, the Nanofitin solutions were additionally injected into a Sartobind STIC nano column (Sartorius) for endotoxin removal. Scale-up manufacturing of recombinant, untagged APL-1030 was performed by Instituto de Biologia Experimental e Tecnológica (iBET, Oeiras, Portugal). *E. coli* DH5α clone-expressing APL-1030 was cultivated in a 30 L Bioreactor (Bioengineering LP351). Initial batch phase consisted of a 15 L culture containing minimal medium. Dissolved oxygen concentration was maintained above 30% saturation by first increasing the stirring, and then by increasing aeration rate. The pH was kept at 7.0 by the addition of base (NH_4_OH, 25%) and the formation of foam was suppressed by automated addition of 20% simethicone emulsion. The exponential feeding phase was started after total consumption of the initial glucose, monitored by an increase in pH. Nutrients were fed using two separate feeding solutions containing glucose, potassium phosphate and trace metals. After approximately 17 h of the fed-batch cultivation mode, APL-1030 expression was induced with 1 mM of IPTG for 3–4 h. Cells were harvested by centrifugation using a Beckman Avanti J-HC.

Biomass (2 kg) was disrupted in APV 2000 homogenizer, and cell debris removed by centrifugation for 45 min at 4000× *g*. Supernatant was clarified by consecutive filtration through 0.8 + 0.45 µm and 0.45 + 0.2 µm filters (Sartopore maxi caps, Sartorius stedim). A purification step was carried out using tangential flow ultrafiltration with a 30 kDa molecular weight cut-off (MWCO) (Sartorius Stedim), the total filtrate was concentrated and diafiltrated by tangential flow filtration using a 5 kDa MWCO membrane (Sartorius). APL-1030 product was further purified by cation exchange chromatography using Fractogel SO3- resin (Merck EMD). The APL-1030 protein was concentrated to ~20 mg/mL by tangential flow filtration (5 kDa MWCO membrane) and loaded onto a Sartobind STIC nano column (Sartorius) for endotoxin removal.

### 2.6. Biolayer Interferometry

Nanofitins were screened in a competition assay to identify candidates targeting an epitope overlapping that of compstatin. Binding kinetic parameters of Nanofitins found to be specific to C3b and representative of each sequence cluster (clusters 1 to 6) were measured by interferometry on the Octet RED96 system (ForteBio, Fremont, CA, USA). Streptavidin biosensors (Sartorius) were first functionalized with biotinylated anti-green fluorescent protein (GFP) Nanofitins [37] (10 µg/mL in TBS containing 0.002% Tween 20 and 0.01% BSA) at 2.0 nm, and then loaded with anti-C3 GFP-tagged Nanofitins (10 µg/mL in TBS containing 0.002% Tween 20 and 0.01% BSA) at 1.5 nm. Biosensors were allowed to equilibrate for 60 s, and binding kinetics were then evaluated by simultaneously exposing biosensors to various concentrations (150, 50, 16.6, 5.55, 1.85, and 0 nM) of C3b in TBS containing 0.002% Tween 20 and 0.01%. Association and dissociation steps were each measured for 180 s. Three cycles of alternating washes for 10 s in 10 mM glycine (pH 2.5) and TBS were used to regenerate biosensors. All steps were performed under continuous 1000 rpm shaking at 30 °C. The biosensor exposed to the 0 nM concentration served as a background reference. Sensorgrams were analyzed using the Octet Data Analysis software 11.1 (ForteBio, Fremont, CA, USA) after a single reference subtraction. A 1:1 binding-fit model was used for fitting. The ratio of the binding response on C3b compared with C3b complexed by a compstatin analogue was used as a measure of the competing potential.

### 2.7. Surface Plasmon Resonance

A single cycle kinetics (SCK) method was used to determine the binding of APL-1030 to C3 and C3b proteins (Complement Technology, Inc.) using a Sierra SPR-32 (Bruker). The SCK method yielded data that fit to a 1:1 Langmuir model with low nanomolar/high picomolar affinities for APL-1030 against both C3 and C3b proteins. Briefly, a new CM5 Biosensor chip from Bruker was docked and conditioned. To test for the binding of the APL-1030, proteins were immobilized sequentially. The conditioned biosensor chip was chemically activated with EDC/NHS. After immobilization, 1 M ethanolamine was injected to block any remaining reactive carboxyl groups in the carboxymethylated dextran surface. The SCK method was used to characterize the binding interactions of APL-1030. For the SCK method, APL-1030 was injected in increasing concentration without regeneration and with shorter dissociation. A 7-point, 3-fold serial dilution assay was performed in duplicate starting at 100 nM for interactions with C3 and C3b. Sensorgrams were analyzed using Sierra Sensors Analyzer software with a double reference to determine the interaction parameters k_a_, k_d_, and K_D_. The reference channel was first subtracted from the ligand channel as the first reference. The internal blank injections were set to closest blank after injection based on the method. The k_d_ was calculated for the two concentrations (with longer dissociation times) by global fitting the off-rate part of the curves. The k_d_ was then set to global constant for fitting rest of the curves. R_max_ was fit locally for each individual curve since the number of available binding sites changed after each injection. Binding data were fit to a 1:1 Langmuir model.

### 2.8. Isothermal Titration Calorimetry 

Isothermal titration calorimetry is described in the Supplemental Methods (Supplemental Information: Methods).

### 2.9. The Wieslab Assay for Alternative and Classical Pathway Inhibition

APL-1030 (iBET) was tested in the commercial Wieslab assay for alternative and classical pathway inhibition according to the manufacturer’s instructions (COMPL AP330, alternative pathway; COMPL CP310, classical pathway [Eagle Biosciences, Amherst, NH, USA). For these ELISA-based assays, Nanofitin solutions were purified in a Sartobind STIC nano column for endotoxin removal. Then, a serial dilution of APL-1030 (0.001–10 µM) was added to diluted, complement-preserved human serum (individual healthy donors represented in n = 3, BioIVT, Westbury, NY, USA). *Serum Handling*: Blood was processed by vendor immediately after collection and centrifuged at 4 °C before being aliquoted and stored at −80 °C. The serum was shipped on dry ice and upon receipt was thawed and aliquoted to single-use vials that were then stored at −80 °C until ready for use. A fresh vial of serum was used for each replicate of the Wieslab assay, and was thawed at 4 °C immediately prior to use. APL-1030 serum dilutions were pre-incubated for 30 min at 4 °C. The serum–APL-1030 mixtures were transferred to precoated plates and incubated for 60 min at 37 °C. Plates were washed 3 times, incubated with conjugated antibody for 30 min at room temperature, then washed an additional 3 times and incubated with substrate for 10–30 min at room temperature. Complement activation was measured by optical density (OD) at 405 nm of a neoepitope on the terminal complement complex C5b-9 using a SpectraMax M5 ELISA reader (Molecular Devices, San Jose, CA, USA). Species cross-reactivity was tested in the Wieslab assay using sera from the following species: cynomolgus monkey (BioIVT, NHP01SRMUN5), African green monkey (BioIVT, NHP03SRMUN5), pig (BioIVT, PIG00SRMUN5), New Zealand White rabbit (BioIVT, RAB00SRMUN5), Sprague Dawley rat (BioIVT, RAT00SRMUN5), and CD-1 mouse (BioIVT, MSE00SRMUN5).

### 2.10. Differential Scanning Calorimetry

The thermal stability of APL-1030 was evaluated for 4 weeks (28 days) at 40 °C. Samples of APL-1030 (1 mg/mL), as received from the supplier, were incubated at 40 °C in 0.5 mL Eppendorf tubes (Hamburg, Germany) for 0 to 4 weeks. Differential scanning calorimetry was performed on a MicroCal VP-Capillary DSC System (Malvern Panalytical, Malvern, UK). Scans were performed at a rate of 60 °C/h from 20 to 110 °C. Thermograms were evaluated by PEAQ-DSC Software version 1.20 (Malvern Panalytical) and qualitatively compared.

### 2.11. Crystallization, Data Collection, and Structure Determination

APL-1030 (iBET, 18.9 mg/mL, 11.5 μM) was mixed 2 to 1 (11.4 μM APL-1030 to 5.7 μM C3b) with C3b (1 mg/mL, 5.7 μM [Complement Technology]). The mixture was then concentrated using an Amicon^®^ Ultra-4 centrifugal filter with a 3 kDa molecular weight cutoff [EMD Millipore, Burlington, MA] and purified by size-exclusion chromatography (Superdex^®^ S200 10/300 GL column [GE Healthcare, Chicago, IL, USA]). The resulting assembly was concentrated using an Amicon Ultra-0.5 centrifugal filter with a 30 kDa molecular weight cutoff [EMD Millipore]. Screens were set up on SWISSCI Midi crystallization plates (SWISSCI AG, Zug, Switzerland). Crystals formed in 100 mM HEPES (pH 7.5), 20 mM magnesium chloride, and 22% polyacrylic acid 5100 sodium salt; these conditions were further optimized in a broad grid screen and additive screen. Crystals were harvested, treated with the same crystallization formulation but supplemented with 20% (*v*/*v*) glycerol as cryoprotectant and flash-frozen in liquid nitrogen.

The X-ray diffraction data were measured at the beamline BL13-XALOC, ALBA, Spain, using a wavelength of 0.97926 Å and a Dectris Pilatus 6M detector. Data reduction was carried out using XDS [39] and AIMLESS [40], as detailed in Table A2. The structure was determined by molecular replacement using PHASER [41] and the publicly available structures of C3b (Protein Data Bank [PDB] accession ID 2i07). APL-1030 was then built in the vacant elements of electron density using COOT [42] and Sac7d peptide (derived from PDB accession ID 1xyi) as a template. Refinement was carried out using Refmac5 [43] as implemented in CCP4 [44], as summarized in Table A3. The final co-structure has been deposited with RCSB as entry 7TV9.

## 3. Results

### 3.1. Discovery of APL-1030

#### 3.1.1. Isolation of Nanofitins Targeting C3b

**Figure A1:**
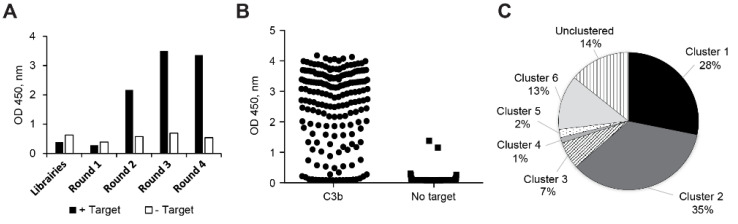
(**A**) Nanofitin (in pAFG12) enrichment by ribosome display, (**B**) ELISA screening for C3b binders, and (**C**) proportion of Nanofitin sequences grouped into clusters based on the homology of their binding site. OD, optical density.

The conversion of C3 into C3b comes with significant structural changes. To facilitate the isolation of Nanofitin hits targeting a structurally conserved region between C3 and C3b, Nanofitin libraries were challenged for four rounds of ribosome display using C3 as a bait for the first two rounds and then alternatively C3 and C3b for the last two rounds. The enrichment of the naïve libraries toward binders was monitored by ELISA using the DNA pools collected at the exit of each round. Specific binding signal of each pool was observed from round 2 and was equivalent on C3 and C3b (Figure A1A). In total, 190 isolated clones were screened by ELISA using crude bacterial supernatant as analytes and immobilized C3b as a target, which revealed a high proportion of positive specific binders (Figure A1B). Subsequent sequencing showed a large sequence diversity, with very few repeated sequences. The Nanofitin sequences could be segregated into six different clusters based on the homology of their variable domain. A strong enrichment for specific sequence patterns was observed as highlighted by the over-representation of the hits within clusters 1 and 2 (Figure A1C). 

#### 3.1.2. Identification of Compstatin-like Nanofitins

**Figure A2:**
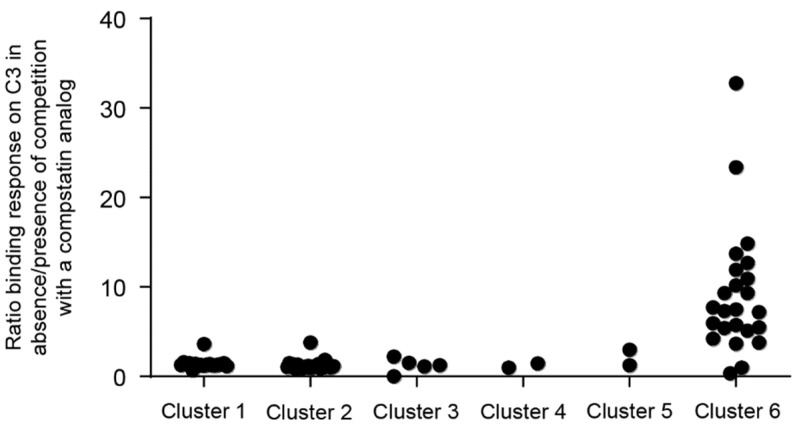
Nanofitin (in pAFG12) competitive binding of compstatin analogue by biolayer interferometry.

The Nanofitins were screened in a competition assay by biolayer interferometry to identify the ones that target an epitope overlapping with that of compstatin. The binding response resulting from the interaction of the different Nanofitins immobilized on a modified streptavidin biosensor with C3b (250 nM) only and C3b (250 nM) complexed by a compstatin analogue (500 nM) was measured. The ratio of the binding response was used as a measure of competing potential (Figure A2). The binding response of the purified Nanofitins from clusters 1 to 5, as measured by biolayer interferometry, was not affected by the presence of the compstatin analogue (ratio of C3b only to C3b complexed by a compstatin analogue of ~1), which suggests that they bind C3b on a different epitope than compstatin. Nanofitins from cluster 6 showed a reduced binding response in presence of the compstatin analogue (ratio > 1), suggesting that the compstatin masks their epitope.

**Figure 2:**
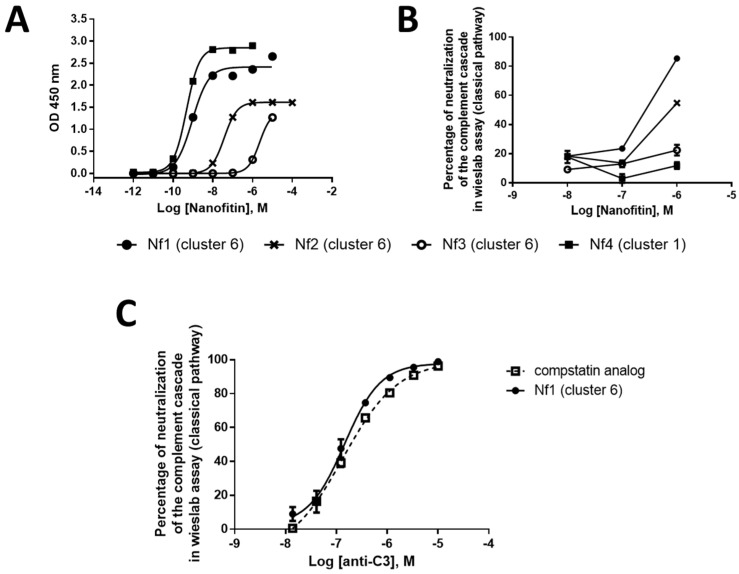
Nanofitin (in pAFG48) (**A**) binding dose–response relationship by ELISA (n = 1), (**B**) complement cascade inhibition by the Wieslab assay of classical pathway functional activity (n = 1), and (**C**) Nf1 complement cascade inhibition compared with a compstatin analog by the Wieslab assay (n = 1). ELISA, enzyme-linked immunosorbent assay; Nf, Nanofitin.

Further characterization included an evaluation of the binding dose–response relationship by ELISA and a screen of complement cascade inhibition using four concentrations of Nanofitin (10, 1, 0.1, and 0.01 µM) in a Wieslab assay of classical pathway functional activity (Figure 2A,B). The clones Nf1, Nf2, and Nf3 from cluster 6 could inhibit classical pathway activation of the complement cascade, with a neutralization potential that is correlated with their EC_50_ by ELISA, with the lowest EC_50_ found for Nf1 (0.92 nM). Of note, Nf4 was not potent at neutralizing the complement cascade, despite a similar EC_50_ (0.47 nM) to Nf1, suggesting that engaging C3 is not sufficient to provide a neutralizing activity. Moreover, Nf1 attained a similar level of neutralization of the complement cascade activated via the classical pathway compared to the compstatin analog (Figure 2C). Taken together, these data suggest that clones from cluster 6 can provide a compstatin-like mechanism of action for complement cascade inhibition by targeting a similar epitope and providing similar neutralization activity. As a result of this screening phase, we chose to continue our development using Nf1 as a hit compound and to potentialize its neutralizing activity by improving its affinity for C3 and C3b.

#### 3.1.3. Affinity Maturation of Nf1

Affinity maturation of Nf1 was performed in two steps. First, a full alanine scan of the binding site was performed to identify the critical residues involved in the specificity for C3. Second, the residues that were not found to be critical were randomized again, and the resulting library was screened by ribosome display with a pressure of selection directed to the k_off_. 

**Figure 3:**
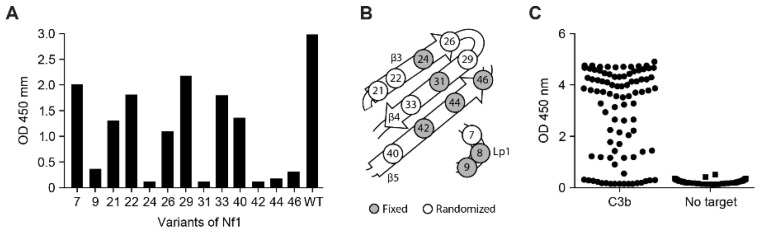
(**A**) Binding capacity on C3b of Nf1 alanine variants at 100 nM in ELISA (n = 1), (**B**) schematic Nf1 residues randomized for affinity maturation, and (**C**) ELISA screening of the variants of Nf1 resulting from the affinity maturation (n = 1). Nf, Nanofitin; OD, optical density.

A total of 13 single alanine mutants of Nf1 were generated, covering all the residues initially randomized in the naïve Nanofitin library, except for position 8, which appeared to already be an alanine. The binding capacity of these different variants was evaluated by ELISA at 100 nM, which corresponded to 10 times the maximal effective concentration (EC_100_) of Nf1 (Figure 3A). In this condition, the alanine variants with a mutation at positions 9, 24, 31, 42, 44, and 46 showed a weak or absent ELISA response (OD_450_ < 0.5), while the other alanine variants retained significant binding capacity (OD_450_ > 1) (Figure 3A). This suggests that the determinant residues contributing to the specificity of Nf1 for C3 are located at the positions 9, 24, 31, 42, 44, and 46. In the focused library for Nf1 affinity maturation, residues found tolerant to the mutation into an alanine were randomized: positions 7, 21, 22, 26, 29, 33, and 40 (Figure 3B).

**Figure 4:**
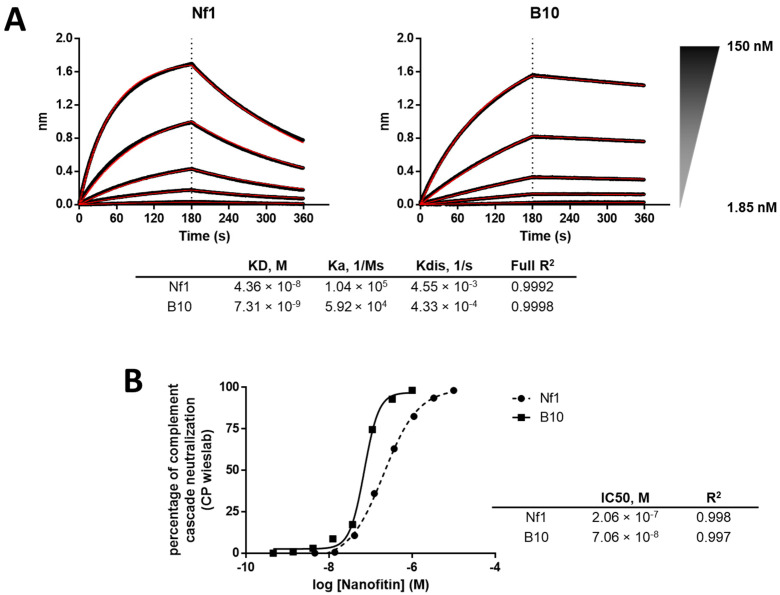
(**A**) Comparison of binding profiles in BLI between Nf1 and the matured B10 variant (i.e., APL-1030) and (**B**) comparison of neutralization potency of Nf1 compared with the matured B10 (n = 1).

The focused library was subjected to three rounds of ribosome display, with selection criteria focusing on the off-rate for the last two rounds. In total, 94 isolated clones were screened by ELISA using crude bacterial supernatant as analytes and immobilized C3b as a target (Figure 3C), showing a strong proportion of specific positive binders. Positive clones were purified and further screened according to three parameters: higher affinity than Nf1 toward C3 and C3b in bio-layer interferometry (BLI), higher neutralization activity in the CP Wieslab assay than Nf1, and conserving a competitive binding with compstatin. The B10 variant of Nf1 was confirmed by BLI to exhibit a slower off-rate and an overall higher affinity compared with Nf1 itself (Figure 4A). As expected, the increase in affinity correlated with an increase in the neutralization potency in the CP Wieslab assay (Figure 4B). This gain in affinity was consistent across assays, including biolayer interferometry, surface plasmon resonance, and isothermal titration calorimetry (Figure 4A, Table 1 and Table A1). This Nanofitin variant that displayed the most optimal pharmacologic and biophysical properties (B10) was selected for further characterization and registered internally as APL-1030.

#### 3.1.4. In Vitro Characterization of APL-1030

The binding affinity of APL-1030 to C3, and separately to C3b proteins, was measured by surface plasmon resonance using an SCK method. Briefly, APL-1030 was injected with increasing concentrations into channels with human C3 or C3b protein immobilized on the biosensor chip. The sensorgrams obtained were analyzed to determine the dissociation constant (K_D_), and APL-1030 was shown to bind to C3 and C3b with affinities of 1.59 and 1.11 nM, respectively (Table 1). These results confirmed the high-affinity target engagement imparted by our screening strategy to identify Nanofitin variants with a slow off-rate. 

**Figure 5:**
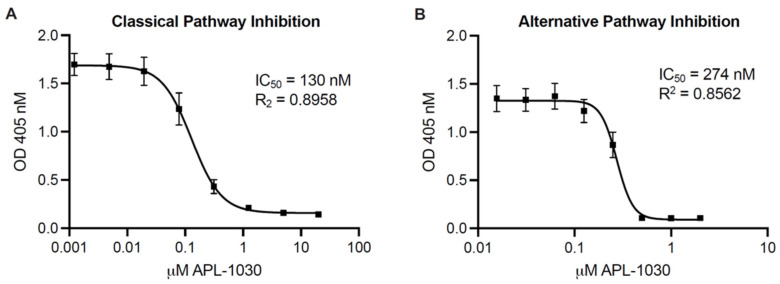
APL-1030 inhibits classical and alternative complement pathway activation. In vitro complement inhibition was assessed by the Wieslab assay for classical pathway activation (**A**) and alternative pathway activation (**B**). IC_50_, half-maximal inhibitory concentration; OD, optical density. Plots represent three independent experiments using 3threedifferent sources of serum from healthy human donors; error bars represent the SEM; lines represent nonlinear regression analysis.

**Figure 6:**
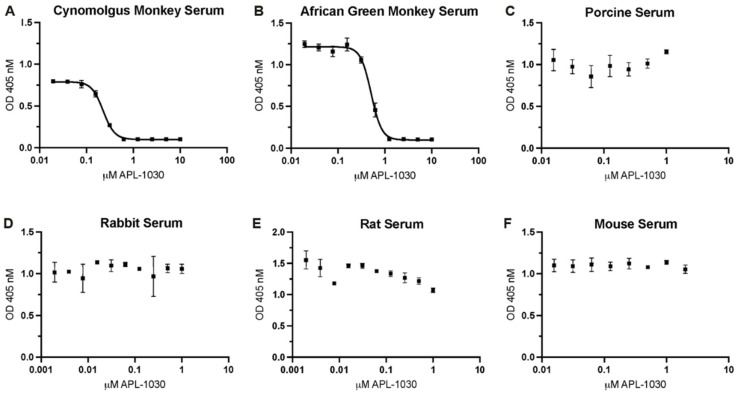
APL-1030 inhibits complement activation in sera from primate species (but not from non-primates). In vitro complement inhibition was assessed by the Wieslab assay for alternative pathway activation by lipopolysaccharides in (**A**) cynomolgus monkey, (**B**) African green monkey, (**C**) porcine, (**D**) rabbit, (**E**) rat, and (**F**) mouse sera. Of the species tested, only cynomolgus monkey (228.2 nM) and African green monkey (511.5 nM) showed measurable inhibition by APL-1030 in the Wieslab assay (**A**,**B**), while non-primate sera (**C**–**F**) showed no inhibitory activity up to 1 μM of APL-1030. All data points in duplicate. IC_50_, half-maximal inhibitory concentration; OD, optical density; error bars represent the SEM; lines (**A**,**B**) represent nonlinear regression analysis.

To assess complement inhibitory potency, APL-1030 was evaluated for in vitro classical and alternative complement pathway inhibition in an ELISA-based assay (Figure 5A,B). Since the lectin pathway uses the same convertase as the classical complement pathway, individual assessment of the lectin pathway was not performed, as this would have been redundant in the context of complement inhibition by targeting C3 and C3b. APL-1030 inhibited classical and alternative pathway activation in human serum with half-maximal inhibitory concentration (IC_50_) values of approximately 130 nM and 274 nM, respectively. Additionally, APL-1030 inhibitory activity was limited to the primate sera tested in the assay (cynomolgus and African green monkey; Figure 6A,B) and produced no inhibition of alternative pathway activation at concentrations of up to 1 µM in the non-primate sera (pig, rabbit, rat, and mouse) used in the assay (Figure 6C–F).

**Figure 7:**
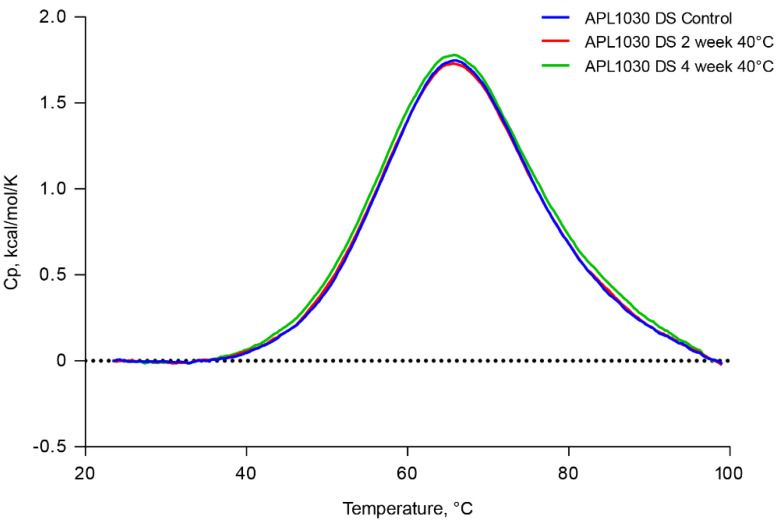
APL-1030 is stable for 1 month at 40 °C. Thermal stability of APL-1030 was assessed by differential scanning calorimetry at time 0, 2, and 4 weeks of constant exposure to 40 °C temperature. Cp, specific heat capacity; DS, differential scanning. N = 1 sample per test condition.

The thermal stability of APL-1030 at 40 °C was assessed out to 1 month using differential scanning calorimetry. Thermograms showed a single specific heat capacity peak at 65 °C and were stable for up to 1 month (Figure 7), demonstrating the high stability of the Nanofitin. 

#### 3.1.5. Structure of the C3b-APL-1030 Complex

**Figure 8:**
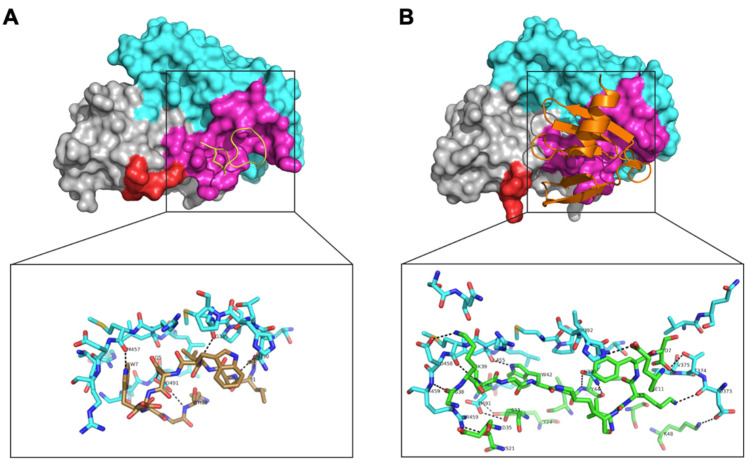
Comparison of the complex structures of compstatin with C3c, and APL-1030 with C3b. The complex structures of (**A**) compstatin, and (**B**) APL-1030, with C3c (PDB 2QKI) and C3b (PDB 7TV9), respectively, with a focus on their MG4 (grey, aa329–aa426) and MG5 (cyan, aa427–aa534) domains. Residues of C3c in interaction with the compstatin are labelled in magenta. A loop (aa370–aa374) subjected to a structural rearrangement between the two complex structures is labelled in red. A comparison of the protein/peptide hydrogen bonds of the binding interface of (**A**) C3c/compstatin with (**B**) is shown in detail.

To determine the binding site of APL-1030 on its target, a crystal structure of C3b-bound APL-1030 was obtained at a spectral resolution of 0.3 nm. The C3b-binding site of APL-1030 fully overlaps with that of compstatin (Figure 8), confirming our discovery strategy to identify candidates with a binding epitope overlapping that of compstatin (PDB accession ID, 2qki). APL-1030 binds to C3b in the same binding pockets as compstatin, which are located at the interface between the MG4 and MG5 domains, but does so through different hydrogen bonds (Table 2). Structural analysis indicated that the peptide residue Trp 42 forms key contacts at the interface of the C3b MG4 and MG5 domains. Additionally, we noted the insertion of the N-terminal β-hairpin of APL-1030 within a cleft in the MG4 domain that is induced by a rearrangement of a loop (residues 370 to 374), which contributes to the larger contact area of APL-1030 (11.2 nm^2^) as compared with the compstatin (5.53 nm^2^). Importantly, the N-terminal end and the C-terminal α-helix of APL-1030 are positioned away from C3b and are potentially available for genetic or chemical conjugation with other functional moieties. 

## 4. Discussion

The objective of these studies was to generate a novel small, high-affinity, compstatin-like inhibitor of complement activation with potential for use in recombinant applications, gene therapies, multifunctional assemblies, and tissue-specific delivery. In this report, we identified and characterized APL-1030 as a novel high-affinity Nanofitin inhibitor of C3-mediated complement cascade activation, which replicates the precedented pharmacology of synthetic compstatin derivatives and allows for the development of novel therapeutics for diseases with high unmet patient need.

Using iterative biochemical processes, we identified Nanofitin binders specific to C3 and C3b, and those which compete with compstatin binding. Affinity maturation of Nf1, the originally identified candidate, improved the C3-binding affinity of the variants. Furthermore, this gain in affinity translated to higher potency compared with Nf1, as demonstrated by Wieslab assays for both the classical and alternative pathways (Figure 4B). 

**Figure A3:**
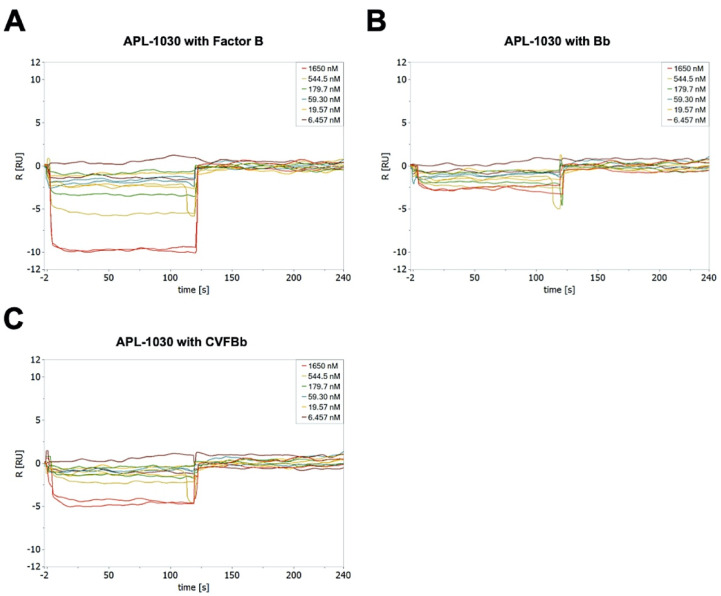
APL-1030 binding to complement components factor B (**A**), the Bb fragment (**B**), and the surrogate convertase, CVFBb (assembled cobra venom factor and Bb fragment, (**C**) by surface plasmon resonance.

APL-1030 exhibited a high binding affinity in the nanomolar range for both C3 and C3b. This suggests that APL-1030 successfully targets both proteins, as C3 undergoes a significant structural change during conversion to C3b [15]. Binding of APL-1030 was specific to C3 and C3b, and did not bind to factor B, the Bb fragment, or the surrogate convertase CVFBb (assembled cobra venom factor and Bb fragment), as assessed via surface plasmon resonance (Figure A3). That binding leads to strong inhibition of both the classical and alternative complement activation pathways. The bioactivity of APL-1030 was expected to be specific to primates based on the targeted binding epitope and previous observations of primate-specific activity of compstatin [45] and compstatin derivatives such as pegcetocoplan (data not shown). This was supported by the lack of response in porcine, rabbit, rat, and mouse serum (Figure 6), further demonstrating specificity for the targeted binding site.

X-ray crystallography demonstrated that APL-1030 binds to C3b in a similar manner to compstatin. Of note, the N- and C-termini of APL-1030 are not involved in binding to C3b and may be available for the construction of multispecific assemblies, enabling additional functionalities such as tissue-specific targeting through receptor binding, half-life-extending moieties, radiopharmaceuticals, and creation of bi- or tri-specific versions for polypharmacology. Indeed, Nanofitins have demonstrated favorable pharmacokinetics and high tolerance for chemical modification (including regiospecific radiolabeling), indicating their potential suitability for diagnostic imaging [46]. APL-1030, as expected based on the hyperthermophillic Nanofitin scaffold, was also thermally stable above body temperature for one month (Figure 7). Together with the self-refolding potential of the Nanofitin technology upon thermal or chemical denaturation, the thermal stability may specifically facilitate use of a broad range of radioisotopes, such as zirconium-89, which forms stable complexes with chelators requiring high complexation temperatures that may limit their use with most protein-based pharmaceuticals [37,47,48]. The unique thermostability of APL-1030 also supports the potential for further development in combination with medical devices and delivery technologies to achieve precision complement inhibition in selected organs.

These findings support APL-1030 as a new targeted C3- and C3b-binding protein, based on Nanofitin technology, which mimics the precedented pharmacology of compstatin and its derivatives, including pegcetacoplan. Because of its recombinant production, unique thermostability, and ability to be used in multispecific assemblies, APL-1030 may eventually broaden the therapeutic choices for patients in high-unmet-need indications, including hematologic, nephrological, ophthalmologic, and neurologic disease areas, who may benefit from inhibition of complement overactivation.

## Figures and Tables

**Table 1:** Surface Plasmon Resonance Results.

APL-1030 Binding to:	k_a_ [1/(M·s)]	k_d_ [1/s]	R_max_	K_D_	X^2^ [RU^2^]
C3	8.94 × 10^4^	1.42 × 10^−4^	NA	1.59 nM	20.15
C3b	8.59 × 10^4^	9.55 × 10^−5^	NA	1.11 nM	14.57

k_a_, association rate; k_d_, dissociation rate; R_max_, maximum theoretical response of the analyte for the ligand level; K_D_, kinetic binding constant; X^2^, resonance units squared.

**Table 2:** Comparison of Protein/Peptide Hydrogen Bonds of C3b/APL-1030 with C3c/Compstatin.

APL-1030	C3b/C3c	Compstatin
	Gly A345 O	Trp 4 N
Lys 5 Nζ	Asp A373 O	
Lys 48 Nζ	Asp A373 Oδ1	
Asp 7 Oδ1	Thr A374 Oγ	
Asp 7 Oδ1	Val A375 N	
Tyr 44 N	Asn A390 Oδ	Ile 1 N, Cys 2 N
Tyr 44 O	Asn A390 Nδ	
Glu 11 Oε1	His A392 Nε2	
Trp 42 Nε1	Leu A455 O	
Ile 40 N	Met A457 O	Trp 7 Nε1
Lys 39 Nζ	Asp A458 Oδ2	
Gly 38 O	Arg A459 N	
Ser 21 Oγ, Asp 35 Oδ2	Arg A459 Nη1	
Tyr 24 OH	Asp A491 N	
Ser 33 Oγ	Asp A491 Oδ2	Gln 5 Nε2
	Asp A491 Oδ1	His 10 N

**Figure A4:**
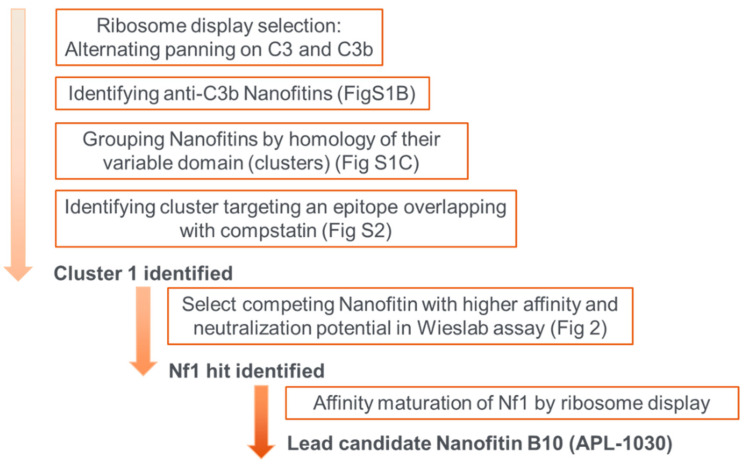
Schematic overview of selection rounds and subsequent steps to obtain the lead candidates.

## Data Availability

Data is contained within the article or Supplementary Material.

## Supplementary Materials

The following supporting information can be downloaded at: https://www.mdpi.com/article/10.3390/biom12030432/s1.

## Appendix A

**Table A1:** Isothermal Titration Calorimetry Results.

APL-1030 Binding to:	N	*K*_D_ nM	Δ*G* kcal/mol	Δ*H* kcal/mol	−*T*Δ*S* kcal/mol
C3	1.18	1.36 ± 1.13	−12.1	−19.3	7.2
C3b	1.21	4.33 ± 0.68	−11.4	−20.2	8.8

N, binding stoichiometry; *K*_D_, binding affinity; Δ*G*, free energy; Δ*H*, binding enthalpy; *T*, absolute temperature; Δ*S*, entropy; ΔG = −RT ln*K*_D_^−1^; ΔG = Δ*H* − *T*Δ*S*.

**Table A2:** X-ray data collection statistics.

Space group	*P* 2_1_
Unit cell parameters	*a* = 99.28 Å, *b* = 104.46 Å, *c* = 102.89 Å α = γ = 90°, β = 97.44°
Resolution (Å)	102–3.4 (3.6–3.4) *
Measured reflections	92,835
Unique reflections	28,812
*R*_sym_ (%)	15.4 (149.2) *
*R*_pim_ (%)	10.2 (100.3)
Completeness (%)	99.2 (98.1) *
I/σI	6.3 (0.9) *
CC(1/2)	0.994 (0.460) *
Redundancy	3.2 (3.1) *

* highest resolution shell of data of 3.6–3.4 Å.

**Table A3:** Crystal structure refinement statistics.

Resolution range	102–3.4 Å		
*R*_work_ *e*	24.0%		
*R*_free_	32.8%		
Protomer details (per asymmetric unit):			
C3b	1e		
APL-1030	1		
rms bond lengths	0.007 Å		
rms bond angles	1.032°		
Ramachandran plot	preferred	allowed	outliers
	76.9%	22.8%	0.4%
