# Supplementary material for: Discovery of APL-1030, a Novel, High-Affinity Nanofitin Inhibitor of C3-Mediated Complement Activation
Source: Biomolecules. 2022 Mar 11;12(3):432. doi: 10.3390/biom12030432 (PMC8946527; doi:10.3390/biom12030432)
Supplement: Supplementary file 1 [file biomolecules-12-00432-s001.zip › biomolecules-1530527-supplementary.pdf]

Supplemental Information: Methods

Isothermal Titration Calorimetry

C3 and C3b (Complement Technology, Inc.) were dialyzed overnight against 1.5 L of 10 mM sodium phosphate pH 7.4 and 150 mM sodium chloride using Slide-A-Lyzer G2 cartridges (1 kDa molecular weight cutoff for C3 and 3.5 kDa molecular weight cutoff for C3b; Thermo Fisher Scientific). Following concentration, final C3 and C3b samples were diluted to 10 μM before use. APL-1030 (iBET) was diluted to 75 μM.

A known amount of C3 or C3b was placed in the sample cell of the isothermal titration calorimetry instrument (Malvern MicroCal ITC200 [Malvern Instruments]). In total, 21 injections of APL-1030 (1.85 μL each) were performed until the final molar ratio was 1.55 to 1 (12.8 μM APL-1030 to 8.3 μM protein). During the experiment, the sample cell and a reference cell were maintained at 25 °C. The amount of power required to maintain that temperature was measured after each injection to quantify the heat generated upon binding. From this, the binding enthalpy (ΔH), binding stoichiometry (N), dissociation constant (K_D), entropy (ΔS), and free energy (ΔG) were calculated.
